# Improving Anti-Neurodegenerative Benefits of Acetylcholinesterase Inhibitors in Alzheimer’s Disease: Are Irreversible Inhibitors the Future?

**DOI:** 10.3390/ijms21103438

**Published:** 2020-05-13

**Authors:** Donald E. Moss

**Affiliations:** Department of Psychology, University of Texas at El Paso, El Paso, TX 79968, USA; dmoss@utep.edu; Tel.: +1-970-629-3927

**Keywords:** Alzheimer’s disease, acetylcholinesterase inhibitor, acetylcholinesterase, butyrylcholinesterase, atrophy, donepezil, rivastigmine, galantamine, metrifonate, methanesulfonyl fluoride

## Abstract

Decades of research have produced no effective method to prevent, delay the onset, or slow the progression of Alzheimer’s disease (AD). In contrast to these failures, acetylcholinesterase (AChE, EC 3.1.1.7) inhibitors slow the clinical progression of the disease and randomized, placebo-controlled trials in prodromal and mild to moderate AD patients have shown AChE inhibitor anti-neurodegenerative benefits in the cortex, hippocampus, and basal forebrain. CNS neurodegeneration and atrophy are now recognized as biomarkers of AD according to the National Institute on Aging-Alzheimer’s Association (NIA-AA) criteria and recent evidence shows that these markers are among the earliest signs of prodromal AD, before the appearance of amyloid. The current AChE inhibitors (donepezil, rivastigmine, and galantamine) have short-acting mechanisms of action that result in dose-limiting toxicity and inadequate efficacy. Irreversible AChE inhibitors, with a long-acting mechanism of action, are inherently CNS selective and can more than double CNS AChE inhibition possible with short-acting inhibitors. Irreversible AChE inhibitors open the door to high-level CNS AChE inhibition and improved anti-neurodegenerative benefits that may be an important part of future treatments to more effectively prevent, delay the onset, or slow the progression of AD.

## 1. Introduction

Alzheimer’s disease (AD) is a severe progressive neurodegenerative disease. Decades of research, hundreds of clinical trials and billions of dollars seeking successful treatment or prevention have been guided by the “amyloid cascade” hypothesis [1], but this effort has produced no interventions that effectively prevent, delay the onset, slow the progression, or arrest AD [2,3,4]. In view of these failures, there is an urgent and unmet need to identify new strategies and focus on other AD-related neuropathological changes, especially those that occur in the earliest stages of the disease, before more advanced irreparable brain damage [5,6,7]. The purpose of this review is to explore the anti-neurodegenerative benefits of acetylcholinesterase (AChE, EC 3.1.1.7) and to suggest irreversible CNS-selective AChE inhibition for improved intervention in AD-related neurodegeneration.

## 2. CNS Neurodegeneration and Atrophy as Major Biomarkers of AD Progression

AD has been traditionally defined on the basis of three classical neuropathological changes in the brains of AD patients. AD was first characterized in the first decade of the last century [8] by the accumulation of extracellular aggregated *β*-amyloid (senile plaque) and intracellular aggregation of hyperphosphorylated tau (neurofibrillary tangles). A third pathophysiological marker of AD is a severe loss of neurons in the midbrain cholinergic system that provides the major cholinergic projections to the cortex and hippocampus [9].

Updated biomarkers for AD, the National Institute on Aging-Alzheimer’s Association (NIA-AA) criteria, include not only measures of *β*-amyloid and tauopathy, but also include markers for AD-associated CNS neurodegeneration and atrophy [10]. In the context of other traditional diagnostic criteria such as cognitive decline, amyloidosis, and tauopathy, CNS neurodegeneration and atrophy correlate strongly with symptoms and risk of cognitive decline and, thus, improve in vivo pathologic staging of the disease [11,12,13,14,15]. 

Investigations of neuronal injury and neurodegeneration, in particular, have led to better surveillance for preclinical signs of AD and identification of the earliest stages of the disease. For example, at the first signs of subtle cognitive decline [16,17], findings of neurodegeneration are 2.5 times more common than amyloidosis [16]. In addition, using neurodegeneration as an early biomarker for AD, cortical atrophy follows a temporal pattern that coincides with cognitive decline [16,18].

## 3. AChE Inhibitors and Anticholinergics Affect Neurodegeneration in AD

Degeneration of basal forebrain neurons causes a loss of cholinergic tone in the basal forebrain cholinergic system, especially projections to the cortex and hippocampus, which is responsible for the severe cognitive losses characteristic of AD [19,20,21,22,23]. The magnocellular neurons of the basal forebrain are among the earliest to undergo severe neurodegeneration in AD [9]. Atrophy of these neurons occurs during normal aging and early in the progression of AD [23,24]. In vivo longitudinal imaging studies indicate that degeneration of the basal forebrain in prodromal AD precedes and predicts entorhinal pathology and memory impairment [25]. Changes in basal forebrain volume is also a reliable indicator of cortical spread of AD-induced neurodegeneration, which supports the contention that basal forebrain neurodegeneration is an upstream triggering event in the development of AD [26]. Atrophy of the basal forebrain, in particular, also predicts cortical amyloid burden [27]. Degeneration of the basal forebrain in preclinical, but cognitively normal suspected prodromal AD, is associated with increased microglial inflammation and amyloid and tau accumulation in vivo at the earliest stages of the disease, which suggests that the loss of central cholinergic tone from the basal forebrain may enable microglial inflammation induced by amyloid and tau accumulation [28]. The cholinergic neurons of the basal forebrain are also among the earliest to show tauopathy, the oligomeric constituents of neurofibrillary tangles in AD [29,30,31,32]. Atrophy of the basal forebrain, in particular, predicts the development of AD in the asymptomatic elderly [33]. Evidence now suggests that the cholinergic cell bodies of the basal forebrain are not completely lost in AD, but that many persist in an atrophied state in which they have lost their cholinergic phenotype [20]. Thus, the collapse of basal forebrain neurons, including loss of their projection fibers and the subsequent absence of their synaptic acetylcholine efflux and cholinergic tone in the cortex and hippocampus, may be a germinal event in the development of AD [20,21,22,23,24,27,29].

The key role of cholinergic tone is confirmed by animal experiments in which basal forebrain lesions (an animal model of AD) or by treatment with anticholinergics (blocking acetylcholine receptors) triggers the formation of *β*-amyloid in transgenic mice [34,35], rats [36], guinea pigs [37], and rabbits [38]. These animal models suggest that all or most normal (non-transgenic) mammalian brains have an incipient age-related capacity to produce amyloid like that which occurs spontaneously in aged primates [39]. Furthermore, the extent of amyloid production occurs on a continuum that is substantially skewed upward toward older animals, those with basal forebrain cholinergic lesions, and those with transgenic with human amyloid-related genes [34,35,36,37,38,39]. The importance of AChE inhibitors, which restore cholinergic function by amplifying the effect of synaptic acetylcholine, is shown by the fact that they are prophylactic against some of these changes [38,40]. 

In humans, the long term use of anticholinergics triggers the accumulation of both plaques and tangles as seen on postmortem examination [41]. Anticholinergics also accelerate the progression from normal cognition to more advanced stages of mild cognitive impairment and conversion into AD-like dementia in elderly persons [42,43,44,45]. In addition, AChE inhibition, an independent effect separate from memantine, slows the clinical progression of AD [46,47,48,49,50,51,52].

The anti-neurodegenerative benefits of AChE inhibition on CNS atrophy, a direct biomarker of AD pathophysiology, are more convincing. For example, in a retrospective analysis patients with mild cognitive impairment, rivastigmine, which inhibits both AChE and butyrylcholinesterase (BChE, EC 3.1.1.8) [53], reduces whole brain atrophy, hippocampal atrophy, and white matter loss [54]. In another study, 20 weeks of treatment with rivastigmine protected against AD-associated white matter loss, an effect that was not observed with donepezil and galantamine, more AChE-selective inhibitors [53]. Rivastigmine-associated protection of white matter is attributed to BChE inhibition [55] and the role of cholinergic signaling, especially involving BChE and its presence in white matter [56,57,58], but rivastigmine is also a potent inhibitor of AChE [53] and such an attribution deserves further study. More specific to AChE, however, randomized, placebo-controlled trials show that short-term donepezil-induced AChE inhibition (one year) in prodromal AD patients slows gray matter atrophy in the hippocampus [59], cortex [60], and basal forebrain [61]. Donepezil-induced AChE inhibition (six months) in patients who have advanced to mild or moderate AD also slows hippocampal atrophy [62]. The anti-neurodegenerative benefits of AChE inhibition on the basal forebrain and its projection areas (hippocampus and cortex) in AD are clear.

The mechanism(s) by which AChE inhibitors produce these disease-modifying benefits are not clear. One hypothesis is that the AChE inhibitors act by enhancing neurotrophic factors, especially nerve growth factor (NGF), which affect key AD-associated pathophysiological processes in the basal forebrain, cortex, and hippocampus [20,63,64,65,66]. The effects of NGF and its possible role in AD, the neurotrophic hypothesis of AD [67,68], and the extensive supporting evidence, have been reviewed in detail elsewhere [20,64]. Briefly, the AD-associated loss of basal forebrain cholinergic neurons, or their cholinergic phenotype, results in a loss of acetylcholine-dependent stimulation of the production and release of NGF from the basal forebrain target tissues (hippocampus and cortex). With declining acetylcholine stimulation, there is a resulting deficit of mature NGF for uptake into the presynaptic terminals of the cholinergic projection axons and inadequate NGF undergoing microtubule retrograde transport back to the basal forebrain cholinergic cell bodies. Without adequate NGF trophic effects, the basal forebrain cholinergic neurons atrophy or lose their cholinergic phenotype [20]. In this scenario, AChE inhibitors amplify acetylcholine-dependent stimulation and release of NGF and, thereby, increase the survival of the basal forebrain cholinergic system, an anti-neurodegenerative effect [20,64]. The role of the basal forebrain cholinergic system, neurotrophic factors, and alternative hypotheses such as tauopathy and inflammation are not mutually exclusive but contribute converging insights into the pathogenesis of AD [65]. Regardless of the mechanism of AChE inhibitor-induced anti-neurodegenerative benefit, there is a call for more effective CNS cholinergic stimulation to improve disease-modifying benefits in AD therapy [20,69].

In summary, increasing cholinergic tone (AChE inhibition) or deceasing cholinergic tone (anticholinergics) produce disease-modifying effects by either slowing or accelerating, respectively, the clinical and pathophysiological progression of AD. In view of the decades of failures of other disease-modifying strategies and the critical need for effective treatments, AChE inhibitors offer an unparalleled opportunity for delaying the onset, slowing the disease, reducing disability and preserving the autonomy of patients at risk for AD.

## 4. Failures of Current AChE Inhibitors

The rationale for the use of AChE inhibitors is to stop the breakdown of synaptic acetylcholine, amplify and extend its impact in the basal forebrain cholinergic system, and to enhance the cholinergic and cognitive functions which deteriorate in normal aging and AD [19,20,21,22,23,70,71,72,73]. While the use of AChE inhibitors has a clear rational basis, their impact on cognitive functions, quality of life, global clinical states, and medicoeconomic benefits are marginal to nonexistent and have fallen short of expectations [74,75,76,77,78]. Even though the currently available AChE inhibitors (donepezil, rivastigmine, galantamine) have produced the most robust anti-neurodegenerative benefits to date [54,55,56,57,58,59,60,61,62], their effects are small and are of more theoretical interest than clinical importance [79,80]. The current AChE inhibitors are far from adequate to meet the demand for highly effective AD interventions [81] that are urgently needed to improve cognitive functions and/or take advantage of the recently recognized additional anti-neurodegenerative benefits [54,55,56,57,58,59,60,61,62].

The main limitation of the current AChE inhibitors is the unavoidable gastrointestinal toxicity that limits their use to doses that are too low to be effective [69]. Direct PET measurements of the maximum in vivo cortical AChE inhibition that can be tolerated in AD patients undergoing donepezil treatment is estimated at ~19% [82], ~27% [83], ~35% [84], and from 28% to 39%, depending on the cortical area [85]. Similarly, in vivo cortical AChE inhibition during rivastigmine and galantamine treatment is estimated at ~28% to 37% [85] and 30% to 40% [86], respectively. This level of AChE inhibition, as found in clinical use, is less than the minimum of ~50% AChE inhibition required for effective AD therapy [69,87,88,89]. In view of these data, it is not surprising that AChE inhibitors produce mainly statistical improvements in cognitive function, but certainly not the powerful clinical improvements that were originally expected [79,80,81]. On the other hand, AChE-induced anti-neurodegenerative benefits are unexpected under such severely limiting circumstances as low levels of inhibition in (25–35%), short-term trials (6 months to one year), and with only a few hundred patients in each experiment [59,60,61,62]. 

It is reasonable to speculate that a broad range of improvement in AChE therapy, high-level AChE inhibition above 50%, could substantially improve anti-neurodegenerative outcomes, but only if the long-time barrier to dose-limiting gastrointestinal toxicity can be overcome [69].

## 5. Mechanisms of Action of Key AChE Inhibitors

The mechanisms by which AChE inhibitors block the catalytic action of the enzyme fall into three major categories: competitive inhibition, pseudo-irreversible inhibitions, and irreversible inhibition. The most important AChE inhibitors used for the treatment of AD are shown in Table 1.

**Figure 1:**
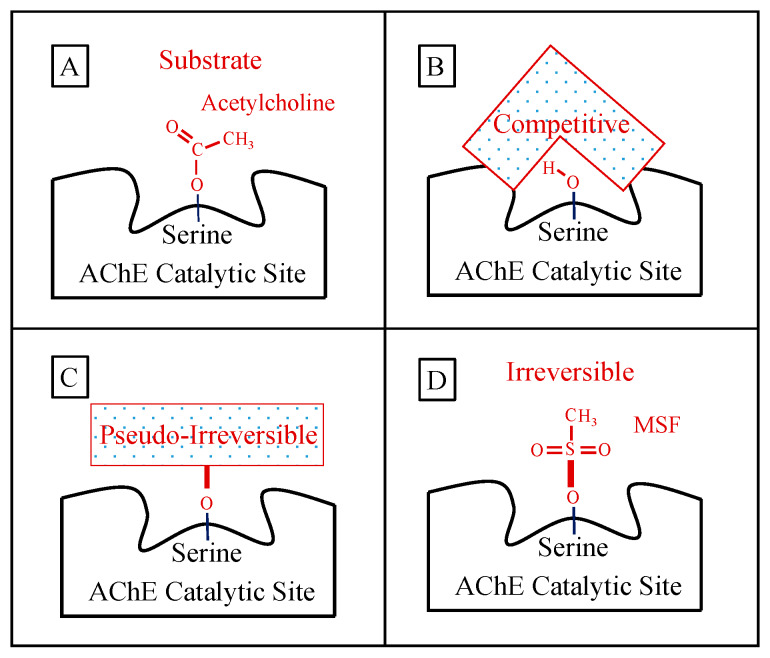
Panel (**A**) shows the normal ephemeral (microseconds) covalent acetyl-enzyme complex that is formed as an intermediate step in the hydrolysis of acetylcholine (shown). Panel (**B**) shows a schematic of a competitive inhibitor binding reversibly (spanning) the catalytic site representing donepezil or galantamine (note that the competitive inhibitor does NOT form a covalent bond with the serine sidechain OH required for acetylcholine hydrolysis). Panel (**C**) shows a longer-lasting covalent bond (signified by heavier red bars) formed between pseudo-irreversible inhibitors (spontaneously hydrolyzed with a half-time of hours) and the enzyme. The schematic box in Panel (**C**) represents the corresponding carbamoyl- or phosphoryl-enzyme covalent binding, respectively, for the case of rivastigmine, metrifonate, or DDVP, wherein the specific molecular structure of each pseudo-irreversible inhibitor intermediate not shown. Panel (**D**) shows an example of the irreversible sulfonyl-enzyme covalent complex (no spontaneous hydrolysis, no recovery) that permanently excludes acetylcholine binding and hydrolysis. The specific sulfonyl-enzyme covalent complex shown in Panel (**D**) is that formed during methanesulfonyl fluoride inhibition.

A schematic representation of how acetylcholine and the key AChE inhibitors interact with the catalytic action of the enzyme is shown in Figure 1:

### 5.1. Mechanisms of Action of Short-Acting AChE Inhibitors—Limited Efficacy

Details of the mechanisms of action of the current AChE inhibitors (donepezil, rivastigmine, and galantamine) have been reviewed in detail elsewhere [79,89]. Briefly, as shown in Table 1, the mechanisms of action of the available short-acting reversible AChE inhibitors fall into two categories: competitive or pseudo-irreversible inhibition. 

#### 5.1.1. Competitive AChE Inhibition: Donepezil and Galantamine

Competitive inhibition is dependent upon the concentration of the inhibitor in the microenvironment of the enzyme in the synapse and the degree to which the inhibitor occupies the catalytic site. It is readily reversible with declining in vivo inhibitor concentration, and, therefore, the duration of the inhibition by donepezil and galantamine is dependent on the rate of inhibitor elimination [79,89].

Donepezil (FDA 1996, Aricept^®^) is a mixed competitive/noncompetitive inhibitor that binds to and orients over the catalytic gorge as well as spans a peripheral binding site which both directly and indirectly blocks catalytic action [90,91]. Donepezil disappears from blood with an elimination half-time of 76 h [88,89].

Galantamine (FDA 2001, Reminyl^®^) is a strictly a competitive inhibitor of AChE [92] that has an elimination half-time of 5–7 h [93]. It was likely the AChE inhibitor antidote in Homer’s Moly (*Galanthus nivalis*) that helped Odysseus rescue his crew from Circe’s malignant anticholinergic posset (*Datura stramonium*), which probably induced the central anticholinergic syndrome (stramonium poisoning), thousands of years ago [94]. Besides inhibiting AChE, galantamine is also an allosteric modulator of nicotinic acetylcholine receptors [95,96], which may lend it some clinical advantages [97].

#### 5.1.2. Pseudo-Irreversible Inhibition: Rivastigmine and Metrifonate 

Rivastigmine (FDA 2000, Exelon^®^) is classified as a pseudo-irreversible inhibitor because it reacts with the critical active site serine to form a covalent carbamoyl-AChE complex that precludes its catalysis of acetylcholine (Figure 1), but the inhibition is short-lived. The duration of rivastigmine-induced inhibition depends on the stability of that bond in the inactive carbamoyl-AChE complex [98]. Although the free drug molecule of rivastigmine is eliminated from the blood with a half-time of about 2.5 h, the covalent bond persists much longer, slowly undergoing spontaneous hydrolysis [98,99] so that rivastigmine-induced AChE inhibition persists for a period of ~8.5 h [88,89,100]. In addition, unlike donepezil and galantamine which are AChE-selective, rivastigmine also inhibits BChE with a duration of 3.5 h [89]. Inhibition of BChE has been proposed as an advantage depending on patient characteristics and genotype [101,102,103], especially for subcortical dementias [104], but BChE inhibition may also affect a range of non-neural functions and toxicities [105,106].

Metrifonate (BAY-A-9826, ProMem, 1997), an organophosphate, was introduced as an AChE inhibitor for the treatment of AD [107,108]. It has been described as a long-lasting cholinesterase inhibitor [99]. Metrifonate, introduced in humans as an acute treatment for schistosomiasis [109], undergoes in vivo spontaneous non-enzymatic rearrangement to 2,2-dicholorvinyldimethyl phosphate (DDVP, dichlorvos) [110,111] with a half-time of ~6 h at pH 7.0 [111,112]. Metrifonate and DDVP both inhibit BChE and AChE [113,114,115]. Most of the cholinesterase inhibition after in vivo administration is due to DDVP [111]. However, the phosphonyl-enzyme covalent bond between DDVP and the catalytic site of CNS AChE (Figure 1) in vivo undergoes spontaneous hydrolysis that results in a reactivated enzyme with a half-time of ~3–4 h [114,116]. Due to the ready hydrolysis of the covalent bond in vivo and resulting enzyme reactivation, metrifonate is most correctly characterized as a pseudo-irreversible inhibitor. Like other short-acting inhibitors, including rivastigmine, it showed little efficacy in treating dementia [107,108]. However, DDVP under its various names is well known to cause organophosphate-induced delayed neuropathy, a late-appearing toxicity that is not related to cholinesterase inhibition, [117,118,119,120,121] and is also a potent inhibitor of cytochrome oxidase [122]. Metrifonate was abandoned as a treatment for AD because it produces severe muscular and life-threatening respiratory paralysis in some AD patients, a sign of organophosphate-induced delayed neuropathy [108,120].

#### 5.1.3. The Failure of Competitive and Pseudo-Irreversible Inhibitors

The fundamental and, so far, insurmountable problem with the current AChE inhibitors in either the clinical management or disease-modifying effects in AD is that there is no discoverable difference between the molecular architecture of CNS and peripheral AChE catalytic sites that has led to successfully identifying an inhibitor for CNS enzyme that does not also inhibit the peripheral enzyme. The result of this failure is that potent inhibition of CNS AChE invariably results in overstimulation of essential cholinomimetic mechanisms in peripheral tissues, especially gastrointestinal control which is highly sensitive to AChE-induced overstimulation. Overstimulation of the gastrointestinal tract causes intolerable dose-limiting nausea, vomiting, and diarrhea [87,88,89] and limits CNS AChE inhibition to the ineffective levels [82,83,84,85,86]. The current AChE inhibitors approved for the treatment of AD are not adequate for meaningful relief from AD-induced suffering or for useful medicoeconomic benefits [74,75,76,77,78].

Both clinical efficacy and adverse events induced by AChE inhibitors are dose-dependent [123], which indicates that high-level CNS AChE inhibition (above 50%) [69] will likely improve efficacy if the problem of adverse events can be overcome [69,124]. Increased CNS AChE inhibition, above what is currently available, will also improve the “CSF Cholinergic Index”, an in vivo physiological measure of an improved CNS ratio of AChE inhibition compared to increased choline acetyltransferase in AD patients [125]. However, high-level AChE inhibition (above the currently available inadequate clinical doses) is blocked by ubiquitous gastrointestinal toxicity produced by currently available AChE inhibitors [87,88,89]. High-level human CNS AChE inhibition (above 50%) in AD patients has only been available in one study that showed promising cognitive enhancement [126]. High-level CNS AChE inhibition in the treatment of AD is an important goal that deserves further study [69]. The single most important objective for the full realization of AChE inhibitor-induced cognitive improvements and anti-neurodegenerative benefits is obtaining effective CNS-selectivity [69,126,127]. The short-term competitive and pseudo-irreversible inhibitors have not been able to meet this fundamental requirement.

### 5.2. Mechanism of Action of Irreversible Inhibitors—CNS-Selectivity

#### 5.2.1. Advantages of Irreversible AChE Inhibition

Irreversible inhibition differs from pseudo-irreversible inhibition in the stability of the covalent bond in the inhibitor-enzyme complex. In the case of pseudo-irreversible inhibition, the covalent bond in the inhibitor-enzyme complex is sufficiently weak so that it undergoes spontaneous hydrolysis, which results in complete reactivation of the enzyme to its full original capacity. In the case of truly irreversible inhibition, however, the covalent inhibitor-enzyme complex is sufficiently strong to be refractory to spontaneous hydrolysis and it permanently inactivates the enzyme molecule. The only way enzyme activity can be restored after irreversible inhibition is through de novo new synthesis of the enzyme. Thus, the duration of irreversible AChE inhibition depends on the rate at which new enzyme is being manufactured, the turnover rate, a characteristic of each tissue [127]. The only clinically useful difference between CNS and peripheral AChE to date is the discovery that CNS AChE is replaced at a much slower rate (t_1/2_ ~12 days) than in the peripheral tissues such as the smooth muscle of the gastrointestinal tract, cardiac muscle, and skeletal muscles (t_1/2_ as short as 1 day). This was recognized as an important tissue-specific difference that might be used, for the first time in the history of AD treatment, to produce CNS-selective AChE inhibition [127].

**Figure 2:**
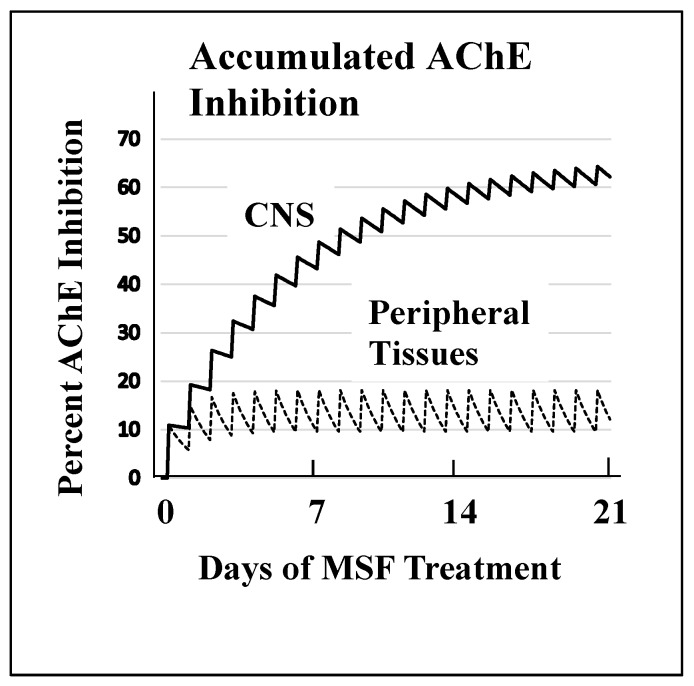
A computational model of the expected accumulated AChE inhibition in the CNS (upper solid line) versus peripheral tissues (lower dotted line) during three weeks of daily doses of an irreversible inhibitor (e.g., methanesulfonyl fluoride, MSF), computed as producing an equal 10% inhibition of currently active AChE in both CNS and peripheral tissues with each dose. The saw-tooth appearance of the lines shows the increment of inhibition (upward points) added with each dose. The downward slope between doses is the decrease in inhibition produced by new synthesis of the enzyme in the dose-to-dose interval. MSF disappears rapidly from blood, within a few hours, producing the pulsatile inhibition shown above. These pharmacological calculations (repeated dosing with recovery between doses) predict the accumulated effects occurring over 21 days [128]. The separation between the levels of CNS versus peripheral tissue accumulated AChE inhibition caused by differences in enzyme recovery rates, as shown above, does not occur with short-acting competitive or pseudo-irreversible inhibitors [69]. (Modified from *Journal of Alzheimer’s Disease*, **55,** Cholinesterase Inhibitor Therapy in Alzheimer’s Disease: The Limits and Tolerability of Irreversible CNS-Selective Acetylcholinesterase, 1285–1294 (2017), with permission of IOS Press.The publication is available at IOS Press through http://dx.doi:10.3233/JAD-160733).The validity of the pharmacodynamics shown in Figure 2 was tested in an experiment in which rats were treated with methanesulfonyl fluoride (MSF), an irreversible AChE inhibitor, in accordance with the 21 day protocol modeled in Figure 2. In this experiment, which is explained in detail elsewhere [128], rats were sacrificed at the end of 21 days of treatment with MSF. As modeled by the computations, CNS AChE was inhibited much more (~75%) than AChE in peripheral tissues (<25% AChE), all without observable signs of toxicity (Figure 3). Seventy-five percent CNS AChE inhibition is at the upper end of the expected therapeutic window for AD and <25% is well below the beginning of toxicity from peripheral tissues [69,87,88,89]. Similarly, rats aged 24 months were pretreated with MSF in a computationally based 4 week protocol designed to produce ~50% CNS AChE inhibition, actually showed in 56% inhibition ex vivo, and such MSF pretreatment enhanced memory function in the aged animals to that equal to young animals [129]. The ability to produce highly selective CNS AChE inhibition without peripheral toxicity has been further confirmed in monkeys (*Macaca fascicularis*) treated with escalating doses of MSF over 3 months, ending with ten weeks of continuous MSF treatment at 5 times the human clinical dose. Cortical biopsies confirmed ~80% and ~45% cortical AChE and BChE inhibition, respectively, with no gastrointestinal toxicity, no neuropathy, nor any other troublesome effects [69].

Figure 2 shows the magnitude of selectivity of an irreversible inhibitor toward CNS AChE inhibition that can be expected from very slow de novo enzyme replacement in the CNS (~12 days) versus fast replacement in peripheral tissues (~1 day). Figure 2 models drug administration given daily for 21 days to approximate a clinically relevant dose of an irreversible AChE inhibitor. These computations, explained in detail elsewhere [69], show that high AChE inhibition (~65%) is expected to accumulate in the CNS because of the slow recovery of activity between doses versus the low expected AChE inhibition (~20%) in peripheral tissues where much of the activity is replaced between doses. The large difference between the rate of de novo AChE replacement in the CNS and peripheral tissues is a key difference that can be exploited to produce highly selective CNS AChE inhibition.

**Figure 3:**
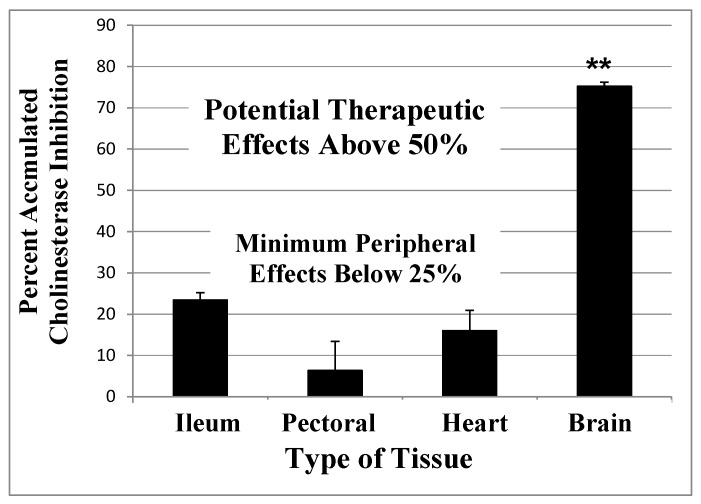
Accumulated AChE inhibition in four rat tissues after three weeks of repeated doses of 0.3 mg/kg MSF (IM) given three times per week to approximate the smaller daily dose shown in Figure 2. The animals were sacrificed 24 h after the last injection and smooth muscle (ileum), skeletal muscle (pectoral), cardiac muscle (heart), and whole brain were assayed for AChE inhibition, compared to untreated controls. CNS is significantly more inhibited than peripheral tissues (**p < 0.01), but peripheral tissues are not different from each other. Error bars show SEM [128]. (From *British Journal of Clinical Pharmacology*, **75**, A Randomized Phase 1 Study of Methanesulfonyl Fluoride, an Irreversible Cholinesterase Inhibitor, for the Treatment of Alzheimer’s Disease, 1231-1239 (2013), with permission Wiley Press).

In summary, Figure 2 and Figure 3 show that an AChE inhibitor with an irreversible mechanism of action given repeatedly over a period of time, similar to a clinical protocol in AD treatment [126], can produce a level of AChE inhibition that is at least double the inadequate 25–35% CNS AChE inhibition observed with the short-acting inhibitors [82,83,84,85,86].

**Figure 4:**
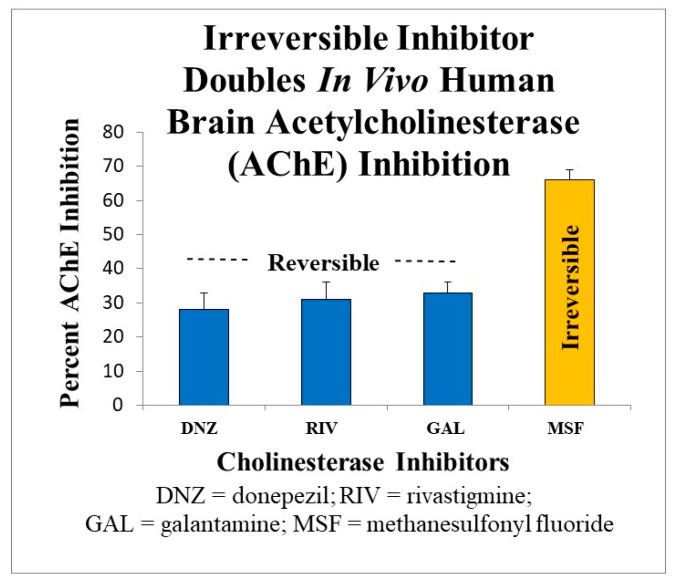
Comparison of brain AChE inhibition produced by reversible inhibitors (donepezil, rivastigmine, and galantamine) to an irreversible inhibitior (methanesulfonyl fluoride). The reversible AChE inhibitors, because of peripheral toxicity, cannot be tolerated by patients at doses that produce more than about 25%–35% AChE inhibition in the brain [82,83,84,85,86]. In contrast, an irreversible AChE inhibitor, because of inherent selectivity for inhibiting brain AChE and the absence of peripheral toxicity, can be administered at doses that produce ~66% brain AChE inhibition [69,126], a level that is within the therapeutic window [87,88,89] and is associated with strong cognitive improvement [126].

Figure 4 shows the direct comparison in CNS AChE inhibition estimated in vivo in Alzheimer’s patients undergoing therapy.

The robust difference between CNS and peripheral tissue AChE inhibition produced by an irreversible inhibitor depends entirely on the difference between the rate at which AChE is newly synthesized in the CNS as compared to peripheral tissues. High-level AChE inhibition can be produced and maintained in the CNS without gastrointestinal toxicity, but only if an irreversible inhibitor is used [69,126].

#### 5.2.2. Sulfonyl Fluorides as AD Relevant Irreversible Inhibitors

Sulfonyl fluorides, including methanesulfonyl fluoride, have been known as irreversible AChE inhibitors since 1954 [130] with a well-understood and solidly irreversible mechanism of action that has been used as a molecular probe of the catalytic site of AChE since the early 1960s [131,132].

The sulfonyl fluorides, like carbamates (e.g., rivastigmine) and organophosphates [133], react covalently with the essential serine oxygen in the catalytic site of AChE to block the enzyme catalytic mechanism (Figure 1) [113,131,132]. The sulfonyl fluorides, specifically including MSF, do not inhibit neuropathy target enzyme, the hypothesized cause of organophosphate-induced delayed neuropathy [134]. Unlike the pseudo-irreversible inhibitors like rivastigmine and metrifonate, however, the sulfonyl-enzyme covalent complex is exceptionally stable and does not undergo spontaneous hydrolysis [131,132], nor can the enzyme be reactivated by strong oxime nucleophilic attack on the covalent bond [135]. Because there is no spontaneous reactivation of the enzyme, the irreversible sulfonyl-enzyme covalent complex was the first tool used to discover that the rate of de novo replacement of CNS AChE activity is more than 10× slower than AChE replacement in peripheral tissues in vivo [127]. Further study of the sulfonyl fluorides indicated that MSF, the smallest and most reactive of the sulfonyl fluorides, was ~100× more biologically active than the larger compounds [136], and the best candidate for the treatment of AD [137].

MSF has uncommon pharmacokinetics. Even though MSF-induced inhibition of CNS AChE disappears with a half-time of ~12 days, the time required for new synthesis, the MSF drug molecule itself is unstable in an aqueous environment such as human blood and undergoes inactivation by in vivo spontaneous hydrolysis to form methanesulfonic acid, an inactive compound, with a half-time of 2.6 h [128,133]. Therefore, MSF administered on a daily schedule like that simulated in Figure 2 produces a pulsatile increment in AChE inhibition that is followed by a drug-free period of ~16 h per day during which new synthesis of uninhibited replacement AChE occurs [69].

The use of MSF for the treatment of AD introduced a special problem. Insofar as MSF is highly selective for the CNS and is free from peripheral toxicity, the optimum dose for patients cannot be determined by increasing the dose until peripheral toxicity is observed, the procedure used for the short-acting AChE inhibitors [87,88,89]. Therefore, the first use of MSF in humans [126] required dose-estimation from the pharmacodynamics calculations shown in Figure 2 [128]. As predicted from the pharmacodynamics calculations and animal experiments (Figure 2 and Figure 3) [69,128], 8 weeks of oral MSF given three times per week to mild to moderate AD patients correctly produced an estimated ~66% CNS AChE inhibition [126], a level of CNS AChE inhibition that is at the upper end of the useful therapeutic window [87,88,89] and which resulted in strong cognitive improvement (~6 points on the ADAS-cog). Furthermore, the MSF-induced cognitive improvement persisted unabated through an additional 8 weeks of placebo [126].

After 8 weeks of placebo treatment, about 5 half-times for the de novo replacement of MSF-inhibited enzyme [69], only ~4% inhibition would remain. Therefore, the duration of strong cognitive improvement over 8 weeks, without further MSF treatment, suggests that MSF produced some long-term benefit that outlasted the direct effects of AChE inhibition. This contention is also supported by an experiment in which MSF treatment also preserves cholinergic neurons and choline acetyltransferase immunoreactivity in the basal forebrain of ischemic rats [138]. These data suggest that MSF-induced AChE inhibition has long-term disease-modifying benefits, perhaps by enhancing acetylcholine-dependent stimulation of NGF production and release and associated basal forebrain survival processes [20,63,64,65,66,67,68].

The high level of MSF-induced CNS AChE inhibition should be equaled by any truly irreversible inhibitor. The CNS selectivity of irreversible AChE inhibitors is due to the slow turnover rate of AChE in the CNS, not a property of the inhibitor molecule beyond the fact that it must form a sufficiently stable inhibitor-enzyme inactive complex that does not undergo spontaneous hydrolysis.

## 6. Discussion

AChE inhibitors address one of the core deficits universally observed in AD, the extensive loss of the basal forebrain cholinergic system and a loss of CNS cholinergic tone that is associated with cognitive loss, suffering and severe medicoeconomic costs. AChE inhibitors have a unique position in the armamentarium of AD in that they offer two different benefits: (1) they directly increase the impact of basal forebrain synaptic acetylcholine on the target tissues associated with cognitive functions; and, (2) they have a less well understood long-term anti-neurodegenerative benefits which include slowing the progression of CNS atrophy and slowing the progression of AD through the clinical stages of dementia. The mechanism(s) for these long-term benefits may be the result of increasing acetylcholine-dependent stimulation of neurotrophic factors [63,64,66], but that determination will require further study.

The anti-neurodegenerative benefits of AChE inhibitors are unparalleled by any other proposed disease-modifying interventions tested to date. The anti-neurodegenerative benefits are evident throughout a wide range of disease advancement from prodromal [54,59,60,61] up through mild to moderate AD [62]. However, AChE inhibitors are expected to have the greatest anti-neurodegenerative impact at the earliest stages of the disease when there is still the maximum possible intact basal forebrain cholinergic system. AChE inhibitor therapy would best start at the earliest appearance of subtle cognitive impairment [15,16,81], a point at which signs of neurodegeneration are the earliest and most common biomarkers of AD and that often precede and predict the accumulation of amyloid [16,17,18]. By extension, it is tempting to speculate that AChE inhibitor therapy could be prophylactic in elderly persons at risk for AD [125,139].

## 7. Conclusions

AChE inhibitors are not likely to be stand-alone treatments, but are likely to be an important part of any future multifaceted drug treatment regimen designed to address different parts of the disease. However, the basal forebrain cholinergic system and acetylcholine are at the nexus of converging well-understood pathophysiological processes in AD, especially neurotrophic-, tau- and inflammation-based hypotheses [20,28,65,140,141,142]. Further development and improvement of CNS-selective AChE inhibition is a direction that deserves further study, especially in view of the anti-neurodegenerative benefits of AChE inhibition and the absence of other successful interventions.

## Figures and Tables

**Table 1:** AChE Inhibitors.

Inhibitor	Mechanism of Action	Additional Notes *
Donepezil	Competitive/Noncompetitive	
Galantamine	Competitive	Upregulates nicotinic receptors
Rivastigmine	Pseudo-Irreversible	Also inhibits BChE
Metrifonate	Pseudo-Irreversible	Induces peripheral neuropathy
Methanesulfonyl Fluoride	Irreversible	High CNS Selectivity

* References to additional notes are found in text.

## Abbreviations

AChE	Acetylcholinesterase (EC 3.1.1.7)
AD	Alzheimer’s disease
ADAS-cog	Alzheimer’s Disease Assessment Scale-cognitive subscale
BChE	Butyrylcholinesterase (EC 3.1.1.8)
CNS	Central nervous system
DDVP	2,2-dicholorvinyldimethyl phosphate (dichlorvos)
FDA	United States Food and Drug Administration
IM	intramuscular injection
MSF	methanesulfonyl fluoride
NGF	nerve growth factor
NIA-AA	National Institutes on Aging-Alzheimer’s Association
